# Can Power-Law Scaling and Neuronal Avalanches Arise from Stochastic Dynamics?

**DOI:** 10.1371/journal.pone.0008982

**Published:** 2010-02-11

**Authors:** Jonathan Touboul, Alain Destexhe

**Affiliations:** 1 Department of Mathematics, University of Pittsburgh, Pittsburgh, Pennsylvania, United States of America; 2 Laboratory of Mathematical Physics, The Rockefeller University, New York, New York, United States of America; 3 Unité de Neurosciences Intégratives et Computationnelles (UNIC), UPR CNRS 2191, Gif-sur-Yvette, France; Indiana University, United States of America

## Abstract

The presence of self-organized criticality in biology is often evidenced by a power-law scaling of event size distributions, which can be measured by linear regression on logarithmic axes. We show here that such a procedure does not necessarily mean that the system exhibits self-organized criticality. We first provide an analysis of multisite local field potential (LFP) recordings of brain activity and show that event size distributions defined as negative LFP peaks can be close to power-law distributions. However, this result is not robust to change in detection threshold, or when tested using more rigorous statistical analyses such as the Kolmogorov–Smirnov test. Similar power-law scaling is observed for surrogate signals, suggesting that power-law scaling may be a generic property of thresholded stochastic processes. We next investigate this problem analytically, and show that, indeed, stochastic processes can produce spurious power-law scaling without the presence of underlying self-organized criticality. However, this power-law is only apparent in logarithmic representations, and does not survive more rigorous analysis such as the Kolmogorov–Smirnov test. The same analysis was also performed on an artificial network known to display self-organized criticality. In this case, both the graphical representations and the rigorous statistical analysis reveal with no ambiguity that the avalanche size is distributed as a power-law. We conclude that logarithmic representations can lead to spurious power-law scaling induced by the stochastic nature of the phenomenon. This apparent power-law scaling does not constitute a proof of self-organized criticality, which should be demonstrated by more stringent statistical tests.

## Introduction

Many natural complex systems, such as earthquakes or sandpile avalanches, permanently evolve at a phase transition point, a type of dynamics called self-organized criticality (SOC) [1], [2]. SOC states are potentially interesting for neural information processing because they represent a state consisting of “avalanches” of recruitment of units as opposed to oscillations or waves. One of the signatures of such critical states is that the size of the avalanches is distributed as a power law, which is particularly interesting for the scale invariance it presents (more precisely, if the probability of observing value x for a given variable is a power-law, 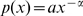, then scaling 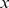 by a constant factor yields to a proportional law: 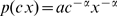). Another notable property is the universality of power-laws in physical phenomena such as phase transitions. In these cases, the exponent is called the critical exponent. Diverse systems show the same critical exponent as they approach criticality, indicating the same fundamental dynamics.

In neuroscience, it is of obvious interest to determine if the recruitment of activity in neural networks occurs in power-law distributed avalanches. This would be evidence that the brain may function according to critical states, rather than the usual wave-type, oscillatory or stochastic dynamics. Moreover, power-law relations are often associated with long-lasting correlations in the system, as with the behavior near critical points. Indeed, the presence of self-organized criticality was inferred for several biological systems, including spontaneous brain activity *in vitro* [3] which displays spontaneous bursts of activity – or “neuronal avalanches” – separated by silences (see also [4] for spontaneous activity in the retina). The distribution of such events was identified to scale as a power law, which was taken as evidence for self-organized criticality in this system (see also review by [2]).

To investigate if criticality is important for brain function, the same type of analysis was also investigated *in vivo*, and in particular in awake animals. However, the difficulty with such analyses is that the activity in awake animals is much more intense compared to *in vitro* [5], with often no visible “pause” in the firing activity, which complicates the definition of avalanches. In a first study on awake cats [6], it was shown that although macroscopic variables such as the extracellular local field potential (LFP) show 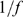 scaling in power spectra, the underlying neuronal activity does not show signs of criticality. In a second, more recent study on awake monkeys [7], power-law scaling was apparent from LFPs when considering the statistics of negative peaks, which are known to be related to neuronal firing. This scale-invariant behavior was taken as evidence for self-organized criticality.

In the present paper, we attempt to resolve these contradictory observations by first performing the same analysis on negative LFP peaks in cats, and using different statistical tests and models to explain these observations. We study the statistical distribution of avalanche sizes, as well as the distribution of the amplitude of negative peaks in the LFPs (linked to neuronal firings), positive peaks, and surrogate data. We then study similar stochastic problems, and investigate whether the results obtained by the experimental data analysis can also be observed in purely stochastic systems without the presence of underlying self-organized criticality. Eventually, we compare the results obtained to the analysis of avalanche data produced by a neural network known to present self-organized criticality [8], [9].

## Material and Methods

### Experimental Data

**Figure 1:**
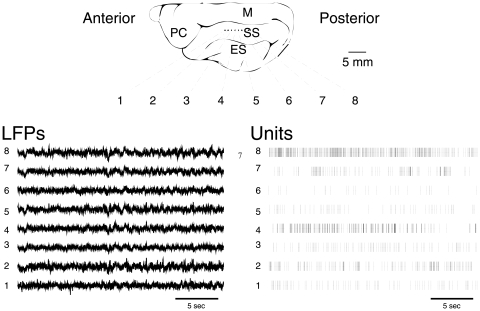
Simultaneous multisite LFP and unit recordings in awake cats. Eight pairs of tungsten electrodes (placement illustrated on top) were inserted in cat cerebral cortex (area 5–7, parietal) as described in detail in [10]. The system simultaneously recorded LFPs (left) and multi-unit activity (right) at each pair of electrode.

The experimental data used in the analysis consist of simultaneous recordings of multisite local field potentials (LFPs) and unit activity in the parietal cortex of awake cats (see Fig. 1), which were obtained from a previous study [10]. A linear array of 8 bipolar electrodes was chronically implanted in the gray matter of area 5–7 of cat cerebral cortex, and the state of the animal was monitored to insure that all recordings were made in awake conditions (quiet wakefulness with eyes-open). Signals were recorded on an eight-channel digital recorder (Instrutech, Mineola, New York) with internal sampling rate of 11.8 kHz per channel, and 4-pole Bessel filters. For LFPs, data were digitized off-line at 250 Hz using the Igor software package (Wavemetrics, Oregon; A/D board from GW Instruments, Massachusetts; low pass filter of 100 Hz). Units were digitized off-line at 10 kHz, and spike sorting and discrimination was performed with the DataWave software package (DataWave Technologies, Colorado; filters were 300 Hz high-pass and 5 kHz low-pass). The data was transferred to LINUX workstations for analysis.

### LFP Analysis

#### Peak detection

**Figure 2:**
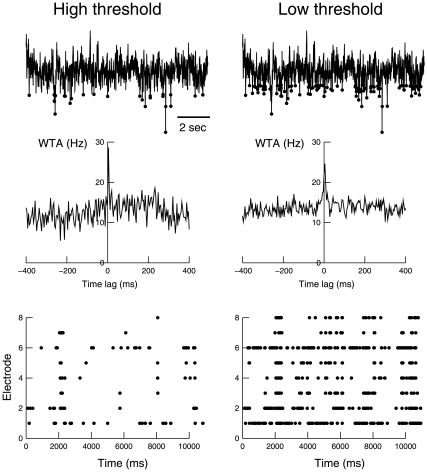
Detection of negative peaks in local field potentials and their relation with neuronal activity. Top: detection of negative LFP peaks. The LFP signal is shown together with the detected nLFPs (circles). Middle: nLFP-based wave-triggered average (WTA) of unit activity, showing that the negative peaks were associated with an increase of neuronal firing. Bottom: rasters of nLFP activity. The same procedure is compared for high threshold (left panels) and low threshold (right panels).

Negative or positive peaks were detected from the LFPs as follows. Signals were mean-subtracted and divided by their standard deviations to obtain comparable amplitude statistics. To avoid artifactual peak detection because of occasional slow components or drifts, the signals were digitally filtered below 15 Hz (high-pass), and the peaks were detected using an adjustable fixed threshold. The peak was defined as the extremum of the ensemble of data points that exceeded the threshold. The detected peaks were then repositioned in the intact original signal (see Fig. 2). The same method was also used for detecting positive peaks.

#### Avalanche analysis

Avalanches were defined by binning the raster of negative peaks of the LFP (nLFPs) into time bins of size 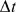 (varied between 4 and 16 ms), and by defining avalanches as clusters of activity among electrodes, separated by silent periods (time bins with no activity), in accordance with previous studies [3], [7]. The “size” of each avalanche was defined as the sum of the amplitudes of all LFP peaks in the avalanche. Similar results were obtained when avalanche size was defined as the total number of peaks within each avalanche (not shown).

#### Surrogate signals

Surrogate signals were generated from the nLFP data sets by shuffling the occurrence times of the different peaks, while keeping the same distribution of peak amplitudes. The occurrence times were replaced by random numbers taken from a flat distribution. The avalanche analysis was then performed on this shuffled data set. Note that, because shuffling changed the timing of the peaks, the whole set of avalanches changed.

### Artificial Data

The results of neuronal avalanche analysis recorded in the cat cerebral cortex will be compared to two types of artificial data sets. From the nature of the LFPs and the links between unit firing and LFP peaks above a certain threshold (see the Results section), we will compare the results of the avalanche analysis of cortical data with two simple stochastic processes (not at criticality) in order to see if the results observed in the avalanche analysis of cortical data can be linked with the stochastic nature of the LFPs. We will also compare the results of the avalanche analysis on experimental data to the avalanche analysis of a network that presents self-organized criticality.

#### Stochastic models

The stochastic processes studied are based on the following two simple models: the shot noise and the Ornstein-Uhlenbeck model.

The first stochastic model considered is a high-frequency shot-noise process consisting of exponential events convolved with a Poisson process. This process, 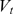, satisfies the equation
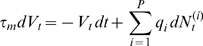
(1)where 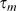 is the characteristic decay time constant of each exponential event, 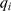 is the jump amplitude of each event, and 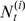 are independent Poisson processes. The solution of Eq. (1) can be written as:
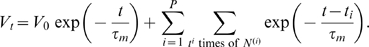
(2)Here, the stochastic variable 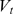 represents the LFP as the summation of a large number of randomly-occurring synaptic events, each described by a decaying exponential.

In the limit of a high number of Poisson processes with summable intensities (or in the limit of a finite number of Poisson process with high firing rate and suitable scaling on the jump amplitude), the solution of equation (1) converges in law towards the solution of the equation:
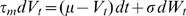
(3)where 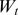 is a Wiener process, 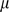 is related to the variables 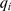 and to the rates of the Poisson processes. This convergence can be proved using for instance Donsker's theorem (see e.g. [11], [12]) and is generally called *diffusion approximation*. The process solution of equation (3) is an Ornstein–Uhlenbeck process, given by:
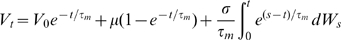
(4)


#### Self-organized critical neural network

We finally performed the statistical avalanche size analysis in a situation where self-organized criticality was known to be present. We used a model proposed by Levina and colleagues, which consists of a network of spiking neurons with dynamical synapses, in which the neuronal avalanches are characterized by a typical and robust self-organized critical behavior [8], [9]. The network is composed of 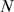 so-called *perfect integrate-and-fire neurons* that integrate random inputs without linear effects such as the cell membrane's leak and without nonlinear effects due to the channels dynamics, and that fire a spike when the membrane potential reaches a fixed threshold. The spike is transmitted with a fixed delay to all postsynaptic neurons with a connectivity weight that varies according to the available reserve of neurotransmitter. This type of network with such dynamic synapses self-tunes to criticality [8].

### Identifying Tail Distributions

#### Power-law and exponential distributions

Mathematically, a continuous random variable 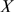 is said to present a power-law distribution if it is drawn from a probability distribution with density:
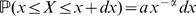
(5)where 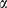 is a constant parameter of the distribution known as the *exponent* or *scaling* parameter, and 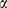 is a normalization parameter. A discrete power-law random variable has a similar, discretized version of the probability, that can be written 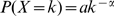. In practice, few empirical phenomena obey power laws for all values of 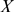, and in general power laws characterize the tail of the distribution, i.e. the probability distribution of values of 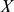 greater than some value 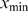. In such cases, we say that the tail of the distribution follows a power law. Moreover, the data often show a truncated power law distribution, i.e. power-law behavior only over a limited range, 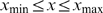.

In this paper, we are interested in discriminating power-laws from another type of distribution: the exponentially-tailed distribution. Random variables with such distributions are characterized for 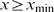 by an exponential probability density, that in the continuous case is given by:
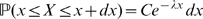
(6)where 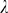 is the parameter of the exponential law and 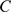 is a scaling parameter. The discrete law can be written in a similar fashion 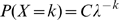. Given some experimental data, the problem is to identify the parameters of the power-law or exponential law that best fits, which means estimating the parameter 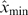, and the power-law exponent 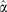 or the exponential-law intensity 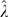.

#### Parameter evaluations

Taking the logarithm of the probability density of a power-law random variable, we obtain 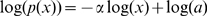. The histogram of the power-law therefore presents an affine relation in a log-log plot. Similarly, the exponential distribution's histogram is characterized by an affine relation in a log-linear plot. For this reason, power-laws in empirical data are often studied by plotting the logarithm (in this paper, when we word *logarithm* and the notation 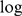 correspond to the natural –neperian– logarithm function) of the histogram as a function of the logarithm of the values of the random variable, and doing a linear regression to fit an affine line to through the data points (usually using a least-squares algorithm). This method dates back to Pareto in the 19th century (see e.g. [13]). The evaluated point 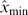 corresponding to the point where the data start having a power-law distribution is mostly evaluated visually, but this method is very sensitive to noise, and is highly subjective (see e.g. [14] and references herein). This widely used technique (and similar variations) generate systematic errors under relatively common conditions (see e.g. [15]). Moreover, there is not any evaluation of the goodness of fit obtained under the power-law assumption. In this paper, we prefer to use a maximum likelihood estimator, which is considered the most reliable of usual estimators (see [15] for a comparison of different estimators). It is known to provide an accurate parameter estimate in the limit of large sample size (see [16], [17]).

Assume that 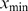, the starting value above which the tail of the distribution, is known, expressions giving the maximum likelihood estimator and maximal likelihood are well known. For the continuous power-law distribution, the maximum likelihood estimator of the exponent parameter 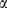 corresponding to n data points 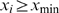 is:
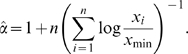
For the continuous exponential distribution, the maximum likelihood estimator of the parameter 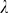 is:
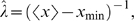
where 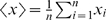 is the average value of the observations 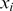.

For the continuous power-law distribution the log-likelihood of the data for the estimated parameter value is:
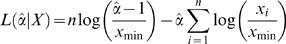
and for the continuous exponential law:
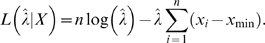
For the discrete exponential distribution, the maximum likelihood estimator has exactly the same expression as that for the continuous exponential law. The exponent estimator for the discrete power-law (truncated or otherwise) has a more complex form than that for the continuous power-law, and cannot easily be expressed as a function of the data (see e.g. [18]). The log likelihood of a sample 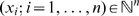 is:
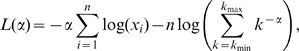
and the estimated value 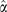 is given by the unique value of 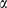 that maximizes the above likelihood function.

Therefore, given the samples 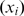 and the value of 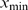 (and possibly 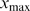), we have expressions for the estimated power-law or exponential parameter. The parameter 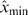 is evaluated then by minimizing the Kolmogorov–Smirnov distance:
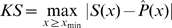
where 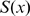 is the cumulative distribution function CDF of the data and 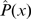 is the CDF of the theoretical distribution being fitted for the parameter that best fits the data for 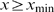), as proposed by Clauset and colleagues in [19].

#### Goodness-of-fit and p-value validation

For a given data set, we now know how to evaluate the best power-law and best exponential-law fits. But is either fit plausible and accurate? In order to answer this question, we use a standard goodness-of-fit test which generates a p-value quantifying the likelihood of obtaining a fit as good or better than that observed, if the hypothesized distribution is correct. This method involves sampling the fitted distribution to generate artificial data sets of size 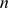, and then calculating the Kolmogorov–Smirnov distance between each data-set and the fitted distribution, producing the distribution of Kolmogorov–Smirnov distances expected if the fitted distribution is the true distribution of the data. A p-value is then calculated as the proportion of artificial data showing a poorer fit than fitting the observed data set. When this value is close to 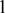, the data set can be considered to be drawn from the fitted distribution, and if not, the hypothesis might be rejected. The smallest p-values often considered to validate the statistical test are taken between 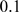 and 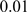. These values are computed following the method described in [15], which in particular involves generating artificial samples through a Monte-Carlo procedure.

#### Direct comparison of models

The methods described above provide the better possible fit for a data set with different probability laws and and the statistical relevance of the model fitted to explain the data set. However, in the case where neither model is rejected by the p-value test, these procedures do not allow to quantify which model provides the better fit.

Several methods have been proposed to directly compare models, such as cross validation [20], fully bayesian approaches [21], minimum description length [22] and the classical log likelihood ratio [23], [24]. The latter, our method of choice, is of particular interest because of the Neyman–Pearson lemma establishing its optimality in certain conditions [25]. This method compares the likelihood of the fit for each model, and selects the model with the greater likelihood. The sign of the log likelihood ratio gives an indication of the model that best fits the data (note that its amplitude in absolute value does not have a direct interpretation), but as other statistical quantities, it is sensitive to noise. The significance of this test therefore needs to be evaluated, and depends on the size of the sample and on the empirical standard deviation of the difference between the log likelihoods of each model (see [24]). This significance test gives a scalar value (also called p-value) between 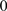 and 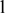. If this value is close to zero, then it is unlikely that the sign of the log likelihood ratio is a result of fluctuations. In that case, it is considered that the sign of the log likelihood ratio is a reliable indicator of which model is the better fit to the data. If it is close to one, the sign is not reliable and the test does not favor either model over the other.

Note that this method compares fits on a given same data set, which requires in particular the use of the same 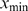 in both models. For this test, we fix 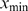 to the mean of the two 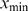 estimated for each law, thereby giving an advantage to the model that fits more of the data.

## Results

### Avalanche Analysis of LFPs from Cat Cerebral Cortex

We start by analyzing the power-law scaling from experimental data. To analyze the power-law relations from LFP activity, we exploited the well-known relation between negative LFP peaks and neuronal firing. We identified the negative peaks of the LFPs (nLFPs), corresponding to events exceeding a fixed threshold, as shown in Fig. 2. The detection was done numerically using a fixed threshold, after digital filtering of the low-frequency components of the signal and the detected peaks were then repositioned in the intact original signal (see Methods). The results of this detection for two different thresholds are displayed in Fig. 2 (top). The detected LFP negative peaks are clearly related to neuronal firing, as evidenced by the wave-triggered average (WTA) of the unit activity. Indeed, the average unit activity presented a clear increase of the discharge probability related to the presence of negative peaks of the LFP (Fig. 2, middle). The same procedure was repeated for all channels, leading to rasters of nLFP activity (Fig. 2, bottom).

**Figure 3:**
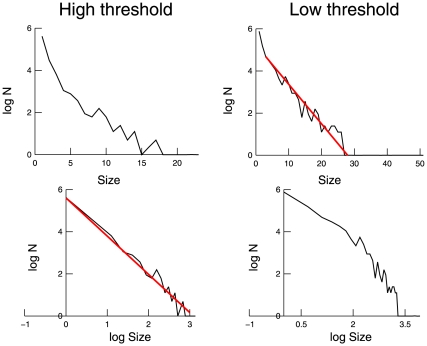
Avalanche analysis of nLFPs in the awake cat. The nLFP avalanche size distributions were computed according to an avalanche analysis (see text). For a high detection threshold, the avalanche distribution is better fit by a power-law (left panels); for a low detection threshold, it is better explained by an exponential distribution (right panels).

We next performed an avalanche analysis based on the occurrence of nLFPs. Similar to previous studies [3], [7], avalanches were defined by detecting clusters of activity across all electrodes, separated by silent periods (see Methods). Fig. 3 shows the distribution of avalanche size (summed amplitudes of all LFP peaks within each avalanche) in log-linear and log-log representations and for two different detection thresholds. For high threshold, the avalanche distribution was better fit by a power-law, whereas for low threshold it was better fit by an exponential distribution. Similar results were obtained when the avalanche size was defined as the total number of events (peaks) within each avalanche (not shown). This shows that the exact functional form of the distribution highly depends on the peak detection threshold. Using a high detection threshold may give the impression of a power-law relation, but lowering the threshold makes the system tend more to an exponential distribution, consistent with the exponential scaling of avalanches calculated from unit activity in the same experimental data [6].

To assess the significance of this result, we performed a Kolmogorov- Smirnov test to the same data. The results of this test are presented in Table 1 for avalanche size defined by the cumulated peak amplitudes. We observe that the distribution of avalanche size is globally well fit by an exponential distribution, which is valid for a large proportion of the data. Indeed, an exponential fit yields significant p-values for both low and high threshold. Moreover, the estimated parameters for exponential fit hardly change when the threshold is varied, again suggesting that the observed exponential fit is meaningful. In contrast, the estimated power-law parameters change significantly when changing the detection threshold, and the low Kolmogorov–Smirnov distance and high p-value obtained for low thresholds correspond to fits of only a small percentage of the data. Thus, although the power-law distribution seems to provide a good fit when only assessed by a linear regression in a log-log representation, this apparent good fit is not supported by the statistical analysis. Instead, the large negative value of the log likelihood ratio, and the very high statistical significance of this test on these data, reveals that the avalanche-size distribution is globally better fit by an exponential distribution.

**Table 1:** Results of avalanche-analysis (summed LFP amplitudes).

Data Type and threshold	Exponential fit				Power-Law fit				Log Likelihood ratio		
	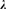	KS	p-val	%	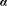	KS	p-val	%	LLR	p-val	Result
Neg. Low	.18	0.028	0.07	38	5.32	0.050	0.94	4	−1211	0.0	Exp
Neg. High	0.13	0.042	0.07	20	2.01	0.077	0	88	−133	0.0	Exp
Pos. Low	0.079	0.052	0	2.7	2.97	0.041	0.70	9	−1275	0.0	Exp
Pos High	0.18	0.033	0.27	31	1.93	0.091	0	93	−351	0.0	Exp

Results of avalanche analysis performed on data obtained from positive (Pos) and negative (Neg) LFP peaks detected with either a Low or High threshold. In this analysis, the avalanche size was the summed amplitude of all LFP peaks within the avalanche. The 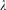 is the estimated exponential parameter, the KS value corresponds to the Kolmogorov–Smirnov distance. The smaller the KS, the better the fit. The closer to 1 the p-value, the better the fit. The % represents the percentage of data explained by the best fit. We observe that the distributions of these data are better represented by exponential fits, and the positive peaks with a low threshold are not well modeled by either an exponential law or a power law. The log-likelihood ratio test always conclude that a better fit is provided by the exponential law. This test is performed over a common range by fitting the data using the same values of 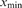 and 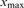 for both laws. The *LLR* value is the value of the log-likelihood ratio. It is negative (positive) if the better fit is the exponential (power-law) distribution. The *p-val* value is the significance log-likelihood ratio (see text). The closer to 0, the more significant the test. *Result* corresponds to the conclusion of the log likelihood ratio test: *Exp* indicates that the log likelihood ratio concludes that the exponential fit is better.

The statistical avalanche analysis performed when the avalanche size was defined as the total number of events (peaks) within each avalanche give an even more ambiguous result. Indeed, both the exponential and the power-law distributions provide a good fit to the data, and the log likelihood indicates that the exponential law provides a better fit but it has a null significance, so does not give any information on the law that best fits the data (see Table 2).

**Table 2:** Results of the avalanche size analysis (number of LFP peaks).

Data Type and threshold	Exponential fit				Power-Law fit				Log-Likelihood ratio		
	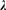	KS	p-val	%	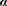	KS	p-val	%	LLR	p-val	Result
Neg. Low	0.19	0.023	0.64	54	1.26	0.020	0.83	18	−77	1.0	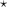 (Exp)
Neg. High	0.27	0.045	0.27	29	1.74	0.009	0.97	100	−61	1.0	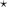 (Exp)
Pos Low	0.23	0.030	0.19	70	1.20	0.021	0.60	54	−232	1.0	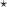 (Exp)
Pos. High	0.36	0.067	0.14	50	1.54	0.012	0.91	100	−110	1.0	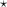 (Exp)

Results of avalanche analysis for avalanche size defined as the number of LFP peaks within the avalanche, for both positive and negative events. Table headers are the same as in Table 1. The 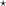 indicates that the fit is not statistically significant.

While these findings suggest that the the nLFP avalanches may also be exponentially distributed, this exponential scaling may be artifactual. Although the underlying neural activity may follow a power-law distribution, the low-threshold condition could add spurious peaks unrelated to neuronal activity, and that would give an exponential trend to the distribution. This increased “noise” is evident in the WTA in Fig. 2, which shows a weaker relation to spiking activity at low threshold compared to high threshold. Thus, additional analyses are needed to determine which of the power-law or exponential scaling is the more closely related to neural activity.

**Figure 4:**
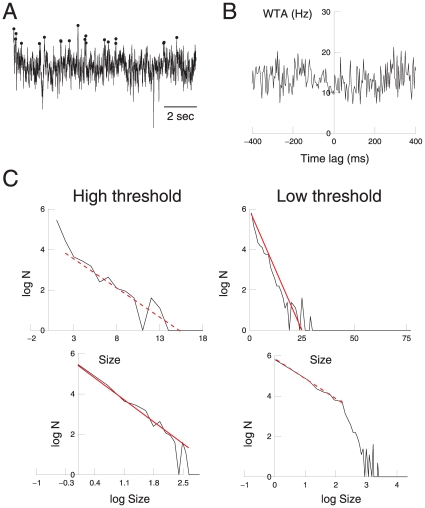
Avalanche analysis of positive LFP peaks in the awake cat. A. Detection of positive LFP peaks using identical procedures as for nLFPs. B. The WTA indicates no relation between pLFPs and unit activity. C. Scaling of avalanche size distribution, showing similar behavior as observed for nLFPs (compare with Fig. 3).

To further test the dependence on unit activity, we have repeated the same avalanche analysis, but using positive peaks of the LFP (pLFP; Fig. 4A). In this case, as expected, the peaks are not related to unit firing (Fig. 4B). Unexpectedly, however, the scaling relations observed in graphical representations for pLFPs are similar to those observed for nLFPs (Fig. 4C): the low-thresholded data fits both a power-law and an exponential law and the high-thresholded data only fits an exponential law. The statistical analysis reveals a power-law for low-threshold pLFPs and an exponential law for high threshold pLFPs. Interestingly, there are also some regions where both the high and low threshold pLFPs distributions display exponential scaling (Fig. 4C, dotted lines). Here, the Kolmogorov–Smirnov test gave results very close to the case of negative peaks. Thus, similar to negative peaks, the apparent good fit of the power-law distribution is not supported by the statistical analysis, as confirmed by the log likelihood ratio test.

**Figure 5:**
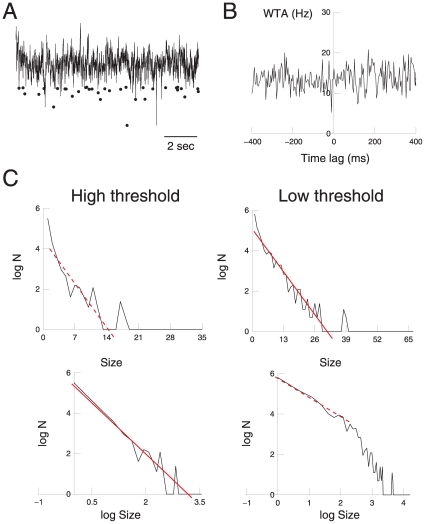
Avalanche analysis of shuffled negative LFP peaks. A. Shuffled peaks obtained from randomizing the timing of nLFP peaks. B. The WTA indicates that shuffling removes the relationship between nLFPs and neural activity C. Scaling of avalanche peak size distribution, showing similar behavior as for nLFPs (compare with Fig. 3).

Another essential test is to generate surrogate data sets. These were produced by taking the nLFP data sets, and randomly shuffling the occurrence times of the different peaks, while keeping the same distribution of peak amplitudes (see Methods). The avalanche analysis was then repeated using these randomized events, and the result is shown in Fig. 5. The shuffling ensures that there is no correlation between these peaks and unit activity, but interestingly, the same relations observed for the nLFPs and pLFPs still persist. In particular, it is quite unexpected that this stochastic system seems to give power-law distributed avalanche sizes. This power-law scaling was seen mostly in the high threshold, while the low-threshold condition behaved more exponentially. The opposite scaling was also seen in restricted regions (Fig. 5C, dotted lines). The statistical tests realized on these surrogate data gave similar results as above (not shown).

**Figure 6:**
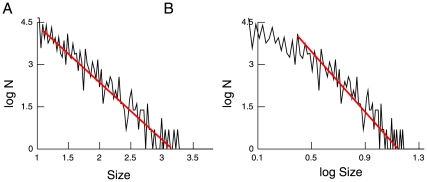
Avalanche-size distributions of negative LFP peaks from single channels. The peak distribution is shown on log-linear (A) and logarithmic scale (B).

The power-law scaling of nLFP size distributions was also apparent when representing graphically the peak distributions from single LFP channels, as illustrated in Fig. 6. To assess the significance of this result, we performed a Kolmogorov–Smirnov test to these data (results are provided in Table 3). For most channels, although graphically we were able to fit the data with a power-law and an exponential distribution, the statistical tests shows that in neither case the fit is statistically significant. For some channels (namely channels 1, 2 and 6), the peak distribution analysis shows, similarly to the multi-electrodes case, that both power-law and exponential distributions provide a good fit to the data, and the log-likelihood ratio test indicates with a high significance level that the data are better fit by an exponential law.

**Table 3:** Results of avalanche-analysis for single-electrode LFP peaks.

Data Type and threshold	Exponential fit				Power-Law fit				Log-Likelihood ratio		
	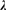	KS	p-val	%	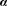	KS	p-val	%	LLR	p-val	Result
Neg. Low	2.39	0.029	0.055	39	6.17	0.056	0.00	33	−47	0.0	Exp
Neg. High	2.82	0.030	0.68	80	9.05	0.048	0.53	34	−4.4	0.04	Exp
Pos. Low	2.07	0.022	0.25	98	6.15	0.041	0.06	26	−37.7	0.0	Exp
Pos. High	2.21	0.038	0.29	56	6.85	0.044	0.10	100	−1.29	0.66	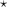 (Exp)

Results of avalanche-analysis for avalanches defined from single-electrode LFP peaks, positive and negative, with low and high threshold. Table headers are the same as in Table 1.

These results suggest that the power-law scaling seen in log-log representations is not necessarily related to neuronal activity, but could rather represent a generic property of these signals. To test this hypothesis, we now turn to the analysis and simulation of stochastic processes.

### Peak Size Distributions from Stochastic Processes

We first investigate computationally whether a power-law relation can be obtained from the peak size distribution of a purely stochastic process. To this end, we generate a high-frequency shot-noise process (as described in Methods), consisting of exponential events convolved with a Poisson process.

**Figure 7:**
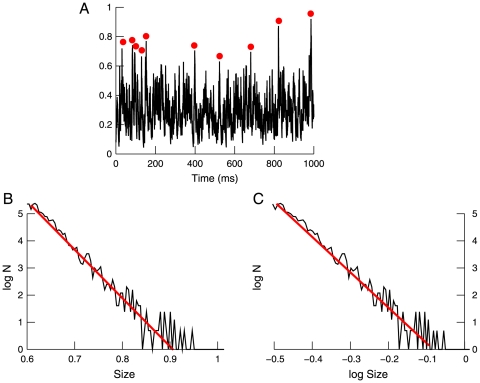
Peak-size distributions for the thresholded Poisson shot-noise process. A. Sample of the stochastic process and detected peaks. B. Peak size distribution on a log-linear scale. C. Same distribution on a log-log scale. Straight lines indicate the best fit obtained using linear regression.

The peaks were detected on the shot noise process 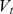 defined by Eq. (2) using a high threshold, in order to mimic the experimental paradigm in Fig. 6A. As for the experimental LFP data, this procedure yielded power-law amplitude distributions, but the same distributions also scaled exponentially (Fig. 7B–C).

#### Peak distribution in the shot noise model

We now investigate this problem analytically. We treat the case where the number of independent Poisson processes 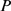 is equal or reducible to one. The case 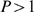 can be treated in the same fashion and yields similar results. In the case 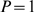, let us denote 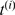 the event times of the Poisson process. T he integrated process (2) simply reads:
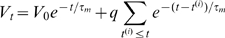
(7)


We are interested in the probability that the supremum of this process reaches a certain threshold value 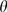 during an interval of times 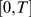. In order to compute this probability, we condition on the number of jumps of the Poisson process in this interval of time, 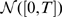. Since the events are disconnected, we have:
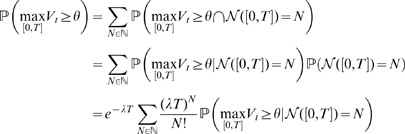
(8)


The maxima of this process occur at the event times of the Poisson process, 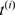, and have the values:
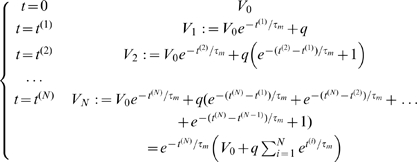
(9)


Furthermore, conditionally on 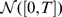 the number of jumps of the Poisson process in the time interval 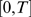, the instants of these jumps are uniformly distributed in the interval 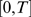. Therefore, the probability that a local maximum is greater than the threshold 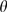 can be written as the following integral:
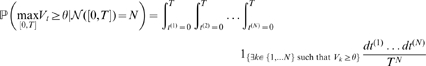
(10)where 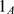 is the indicator function of the set 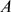. Therefore, the peak distribution we are searching for has the expression:
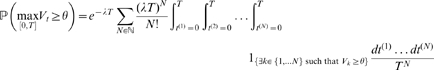
(11)This integral cannot be simplified further, but can be accurately approximated using a numerical integration method and truncating the series. The approximation error is proportional to the rest of the exponential series 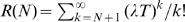.

Let us now consider the distribution of the maxima of the process (7) given that the process does an excursion above a certain threshold. This case can be treated in a similar fashion, but considering the distribution of local minima also. These local minima are reached at times 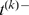 just before the jumps of the Poisson process, and their value are 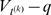. The probability of an excursion above 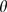 and exceeding 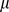 (event denoted 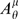) can therefore be written as:
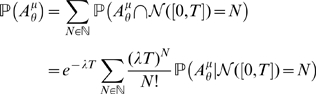
and the probability 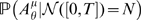 can be easily evaluated numerically using the following representation:
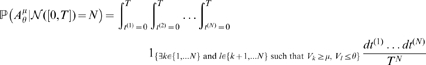
(12)


**Figure 8:**
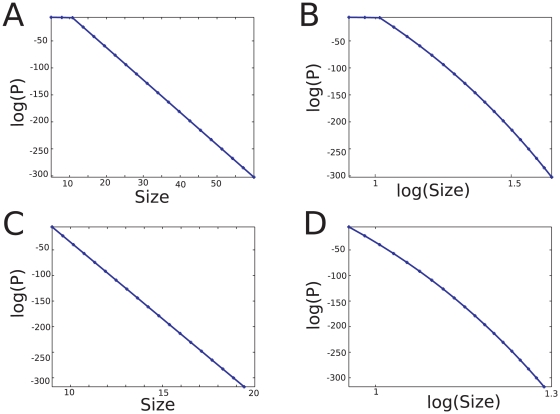
Peak amplitude distribution for the Shot-Noise model. Single-barrier case (A,B) on a log-linear scale (A) and on a log-log scale (B) show a globally linear trend. Excursions (C,D) show exactly the same profile. Simulation parameters: intensity of the process 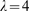, 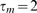, 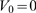, 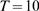, 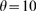, maximal value of peaks considered 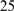 (see text).

Simulation results of these distributions are presented in Fig. 8 and predict the results obtained by numerical simulations in Fig. 7: both exponential and power-law distributions give a good model for the peak amplitude distribution. The results of the statistical analysis are in accordance with this observation, and are provided in Table 4. Indeed, we observe that the exponential distribution gives a good model in both the single barrier and the excursion case, and the power-law distributions provide a good agreement with the computed theoretical distributions only in the excursion case. Note that we did not use the log-likelihood ratio because this statistical test is defined through the computation of the likelihood of a given probabilistic model on a data set, and here we do not have data sets but we directly compute the probability distributions.

**Table 4:** Results of avalanche-analysis for thresholded stochastic processes.

Data type	Exponential fit			Power-Law fit		
	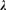	KS	p-val	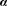	KS	p-val
Shot-Noise	0.70	0.103	0.12	10.08	0.185	0.00
single-barrier						
Shot-Noise	0.72	0.014	1.00	15.00	0.094	0.28
excursion						
Ornstein–Uhlenbeck	2.40	0.042	0.97	44	0.077	0.62
single-barrier						
Ornstein–Uhlenbeck	2.42	0.0051	1.00	48.00	0.012	0.92
excursion						

Results of avalanche-analysis for avalanche-sizes analytically determined for four stochastic processes. Table headers are the same as in Table 1. The estimated power-law is large because we considered the tail of the distribution, and since the data present an exponential trend, the estimated power-law exponent becomes larger when thresholds are high. Even if the p-value is high, the fit is not realistic and the does not hold for larger intervals. We do not use the log-likelihood ratio since it is defined for samples and does not really make sense for distributions.

#### Peak distribution in the Ornstein-Uhlenbeck model

In the case of the Ornstein-Uhlenbeck model, the stochastic process modelling the LFP has the same regularity as the Brownian motion, and therefore is is nowhere differentiable, and has a dense countable set of local maxima. In that case, peaks are no more defined as local maxima of the process, and the problem is reduced to determining the probability that the process exceeds a certain value. This probability can be deduced from the law of the first hitting time of the Ornstein–Uhlenbeck process. Indeed, let us denote by 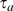 the first hitting time of the threshold 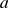 for the Ornstein-Uhlenbeck process given by equation (4). The probability that the process exceeds a certain level 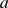, given that it exceeds the threshold 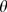, is given by:
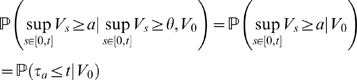
(13)


The excursion case continuous equivalent consists in considering the probability of exceeding a certain quantity 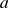 before going back under the excursion threshold 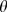. This probability can be written as:
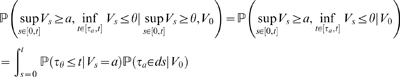
(14)


Therefore, the repartition function of the maxima, and that of the maxima above a certain threshold, can be deduced from the repartition function of the first hitting time of the process 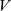. As reviewed in [12], [26], there is no closed form solution for the probability distribution of these hitting times, but they can be efficiently numerically computed. The most convenient solution involves solving a Volterra integral equation to obtain the law of the first hitting time variable (see e.g. [12], [27], [28]).

**Figure 9:**
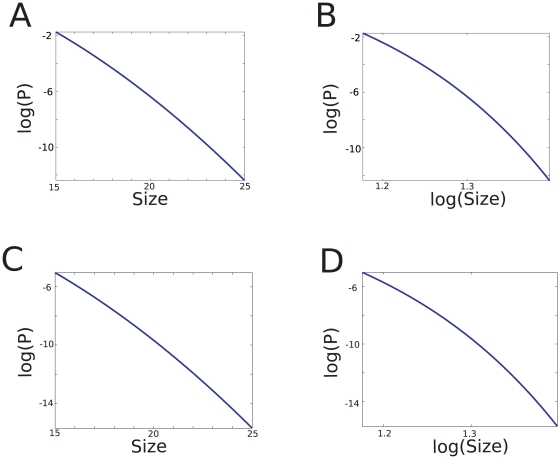
Peak amplitude distribution for the Ornstein-Uhlenbeck process. (A,B): single-barrier peaks, on a log-linear scale (A) and on a log-log scale (B), and excursions (C,D), on a log-linear scale (C) and on a log-log scale (D). Both case present the same profile and a globally linear trend for both axis. Simulation parameters: intensity of the process 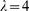, 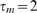, 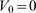, 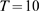, 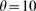, maximal value of peaks considered: 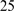 (see text).

In this case again, the same remarks apply: we observe (see Fig. 9) for both the single-barrier and the excursion problems that the peak-amplitude distribution is fit equally well by either a power-law or exponential distribution. This is supported by the more rigorous statistical analysis (see Table 4): both the exponential and the power-law distributions provide a good agreement with the distributions computed numerically form the formulas derived.

### Avalanche Size Distribution in a Neural Network at Criticality

**Figure 10:**
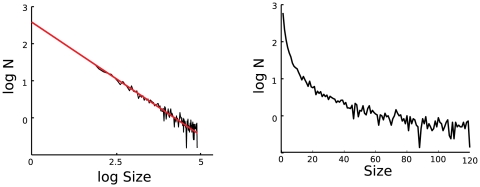
Avalanche analysis of a simulated neural network displaying self-organized criticality. The power-law distribution provides a very good graphical fit (A), whereas the exponential distribution provides a poor fit (B). Data from ref. [8].

We finally performed the above statistical analysis on the avalanche data generated by the artificial network in the critical state of Levina and colleagues [8], [9] (data kindly provided by Anna Levina). The avalanche size distributions obtained are plotted in Fig. 10, and the results of the statistical analysis show very clearly that the data are very well fitted by a power-law in this case (see Table 5). We conclude that in the case of a neural network at criticality, the ambiguity observed in the experimental data is not present, even when using the same number of avalanches as in our data. Thus, this analysis brings another argument to support the absence of robust power-law scaling in the experimental data.

**Table 5:** Results of avalanche-analysis for the artificial network model [8] at criticality.

Data type	Exponential fit			Power-Law fit			Log-Likelihood ratio		
	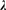	KS	p-val	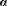	KS	p-val	LLR	p-val	Result
Full data set	0.10	0.2820	0.00	1.44	0.0027	0.85	1645	0.0	PL
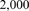 avalanches	0.10	0.2806	0.00	1.42	0.0061	0.80	2483	0.0	PL

Results of avalanche-analysis for avalanche-sizes determined using a sequence of 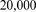 avalanches produced by the artificial neural network model, and a smaller set of 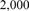 avalanches corresponding to the typical number of avalanches we have in our experimental simulations. The power-law model provides a very good fit, with high p-value, whereas an exponential law is not a good statistical model of the data in either case.

## Discussion

In this paper, we have provided an analysis of multisite LFP recordings in awake cats, using the detection of negative LFP peaks (nLFPs), as done in a previous study [7]. The analysis shows that the occurrence time and amplitudes of nLFPs can show power-law distributions, but in a manner that depends on the detection threshold. High thresholds, which select events of exceptionally large amplitude, tend to give power-law relations. In contrast, low thresholds, which select many events, give rise to exponential distributions, similar to stochastic processes. The application of more rigorous statistical tests, such as the Kolmogorov–Smirnov test, shows that the power-law relations are not supported by solid statistical grounds. The dependence on the threshold is much weaker in the statistical data analysis, as we can clearly see in Tables 1 and 2.

Because the exponential scaling could be interpreted as a spurious result due to the addition of a large number of peaks unrelated to neuronal activity, we considered two controls: first, positive LFP peaks, which are not related to neuronal activity, and randomly shuffled peak times, which makes the system equivalent to a stochastic process with the same peak amplitude distribution as the data. The two cases show similar apparent power-law scaling and dependency to threshold as for nLFPs.

These results suggest that the spurious power-law scaling could be a generic property of thresholded stochastic processes. To investigate this point in more depth, we studied a similar peak detection paradigm applied to two simple stochastic models, one corresponding to LFPs arising from a linear summation of spikes arriving at the times of a Poisson process (a shot-noise process) and the diffusion limit of this phenomenon (an Ornstein–Uhlenbeck process). The former case can be solved in a closed integral form while the latter case is solved using the laws of the first hitting times of the Ornstein-Uhlenbeck process. Both models demonstrate the same ambiguity: when only looking at the log-linear and log-log plots, and both power-laws and exponential laws can be fitted. However, the application of the more rigorous Kolmogorov–Smirnov test demonstrated that some apparent power-law scaling (as seen from log-log representations) is not based on solid statistical grounds, in real data as well as in the theoretical laws computed, in agreement with previous studies (see e.g. [15]).

This analysis therefore confirms that thresholded stochastic processes can display power-law scaling, but only when performing simple line fitting in log-log representations. Indeed, we observe that it is always possible to fit a power-law distribution to the tail of the distribution with a quite good agreement, but these fits do not hold for large threshold values (see Table 4). The estimated laws yielded high values of the exponent which is not very realistic in general. This is consistent with the findings reported above for LFPs: the power-law scaling of LFP peaks displays very similar properties to that of stochastic processes, which supports the idea that experimentally observed power-law scaling is not necessarily related to neuronal activity, but may be explained by a generic property of thresholded stochastic processes.

The same analysis applied to a network presenting self-organized criticality confirms with no ambiguity that the distribution of avalanche size presents a clear power-law distribution, whereas in cortical LFPs the power-law scaling in log-log representations was not supported by statistical analyses. We conclude that power-law scaling, particularly when deduced from log-log representations, does not constitute a proof of self-organized criticality, but should be complemented by more sophisticated statistical analyses.

Thus, contrary to a previous study in monkey [7], where the same controls were not done, our analysis suggests that, in awake cats, the power-law scaling is not related to neuronal activity but is rather an artefact of the thresholding procedure. In agreement with this, a previous analysis [6] failed to see evidence for power-law distributions and avalanche dynamics from spiking activity in the same data set, which rather scaled exponentially. However, there is still the possibility that these differences arise from other factors such as the different species, brain areas, or cortical layers used in these experiments.. Further studies should address these points.
